# Os Desafios da Insuficiência Cardíaca Ontem, Hoje e Amanhã, e os 20 Anos do DEIC

**DOI:** 10.36660/abc.20201200

**Published:** 2021-02-19

**Authors:** Evandro Tinoco Mesquita, Ana Paula Mendes, Lidia Moura, José Albuquerque de Figueiredo, Fabiana G. Marcondes-Braga, Fernando Bacal, Maria da Consolação Vieira Moreira, Nadine Oliveira Clausell

**Affiliations:** 1 Universidade Federal Fluminense Hospital Universitário Antônio Pedro NiteróiRJ Brasil Universidade Federal Fluminense Hospital Universitário Antônio Pedro, Niterói, RJ - Brasil; 2 Pontifícia Universidade Católica do Paraná CuritibaPR Brasil Pontifícia Universidade Católica do Paraná, Curitiba, PR - Brasil; 3 Universidade Federal do Maranhão São LuísMA Brasil Universidade Federal do Maranhão, São Luís, MA - Brasil; 4 Hospital das Clínicas Faculdade de Medicina Universidade de São Paulo São PauloSP Brasil Instituto do Coração (InCor), Hospital das Clínicas, Faculdade de Medicina, Universidade de São Paulo, São Paulo, SP - Brasil; 5 Universidade Federal de Minas Gerais Faculdade de Medicina Belo HorizonteMG Brasil Universidade Federal de Minas Gerais Faculdade de Medicina, Belo Horizonte, MG - Brasil; 6 Hospital de Clínicas de Porto Alegre Porto AlegreRS Brasil Hospital de Clínicas de Porto Alegre - Serviço de Cardiologia, Porto Alegre, RS – Brasil

**Keywords:** Insuficiência Cardíaca/tendências, Envelhecimento da População, Hospitalização, Pesquisa Médica Translacional, Diretrizes, Produção Científica

O Departamento de Insuficiência Cardíaca (DEIC), da Sociedade Brasileira de Cardiologia, completou 20 anos de existência em 2020 e representa um robusto legado nas áreas de atividades científicas e associativismo da cardiologia brasileira.

Sua construção representou um marco importante no enfrentamento da insuficiência cardíaca (IC), uma complexa síndrome clínica progressiva e frequentemente fatal. A IC deve ser abordada de forma multidisciplinar, apoiada na ciência translacional e nas boas práticas (diretrizes e protocolos clínicos), envolvendo pacientes, famílias, cuidadores, gestores e toda a sociedade, tendo em vista seus impactos sociais no Brasil e no mundo.

Com o envelhecimento populacional e o aumento da sobrevida dos pacientes com doenças cardiovasculares, a prevalência de IC está aumentando globalmente, com uma estimativa de 26 milhões de pessoas acometidas no mundo, além de milhares de casos não diagnosticados.^1^ A IC é causa líder de hospitalização no mundo, e isso resulta em uma sobrecarga em todos os níveis de cuidado. Estima-se que a IC afeta aproximadamente 2,5 milhões de pessoas no Brasil, e um estudo recente revelou o seu impacto financeiro no país, com um gasto estimado de R$ 22,1 milhões/6,8 milhões de dólares no ano de 2015.^2^ Além disso, o estudo mostrou uma substancial perda de bem-estar; dos 521.941 de anos de vida perdidos ajustados por incapacidade, ajustados para comorbidades, há 270.806 de anos de vida saudável perdidos em virtude de incapacidade e 251.941 de anos de vida perdidos em decorrência de morte prematura.

O DEIC foi idealizado a partir da liderança da professora Maria da Consolação Vieira Moreira e teve o apoio do professor Gilson Soares Feitosa, na ocasião, presidente da Sociedade Brasileira de Cardiologia (1999-2001), mobilizando líderes de todo o Brasil, com destaque para o professor Edimar Alcides Bocchi, levando à formação do Grupo de Estudos de Insuficiência Cardíaca (GEIC) no ano de 2000. Em 6 de julho de 2001, sob a presidência da professora Maria da Consolação, foi realizado, em Belo Horizonte-MG, o I Simpósio Brasileiro de Insuficiência Cardíaca, por ocasião do XII Congresso da Sociedade Mineira de Cardiologia ( Quadro 1 ).

**Quadro 1:** – Presidentes GEIC/DEIC

Primeira Diretoria GEIC	Presidentes GEIC/DEIC
**Presidente:** Edimar Alcides Bocchi	**2000-2001** : Edimar Alcides Bocchi
**Vice-presidente:** Denílson Campos Albuquerque	**2002-2003:** Edimar Alcides Bocchi
**Secretário:** Fábio Vilas-Boas Pinto	**2004-2005:** Fábio Vilas-Boas Pinto
**Diretora científica: ** Maria da Consolação V. Moreira	**2006-2007:** Nadine Oliveira Clausell
**Membros da Comissão Científica:**	**2008-2009:** Marcelo Westerlund Montera
Evandro Tinoco Mesquita	**2010-2011:** Fernando Bacal
Dirceu Rodrigues de Almeida	**2012-2013:** João David de Souza Neto
Fernando Bacal	**2014-2015:** Dirceu Rodrigues de Almeida
Marco Aurélio Silva ( *in memoriam* )	**2016-2017:** Luis Eduardo Paim Rohde
Nadine Oliveira Clausell	**2018- 2019:** Salvador Rassi
Salvador Rassi	**2020-2021:** Evandro Tinoco Mesquita

Com uma trajetória de sucesso, construída por líderes da IC do país, devido ao crescimento do número de associados e relevância na produtividade científica, o GEIC foi se transformando paulatinamente no DEIC, finalmente criado em 2011, na gestão do professor Fernando Bacal. Esse fato importante ocorreu durante o X Congresso Brasileiro de Insuficiência Cardíaca – ao comemorar 11 anos da fundação do GEIC, na cidade de Belo Horizonte-MG ( Quadro 1 ).

Desde sua criação, são realizados, anualmente, congressos de elevada qualidade científica, com intercâmbio internacional e que proporcionam à comunidade médica brasileira aprimoramento do estado da arte do cuidado multidisciplinar e tratamento da IC. Nos últimos congressos, temos tido mais de 1.000 inscritos e cerca de 200 temas livres têm sido apresentados, possibilitando trocas de experiências com especialistas de várias localidades e nomes de referência, do Brasil e do mundo. Em 2020, devido aos impactos da pandemia do novo coronavírus, o DEIC, de forma revolucionária, realizou um congresso virtual – Heart Failure Summit Brazil 2020 –, com a proposta de apresentar e debater os principais avanços que, nos últimos 12 meses, transformaram a IC e serão motivos para mudanças na nossa Diretriz de IC prevista para ser lançada no primeiro semestre de 2021 ( Quadro 2 ).

**Quadro 2:** – Simpósio e Congressos GEIC/DEIC

**1º Simpósio Brasileiro de Insuficiência Cardíaca**	**X Congresso Brasileiro de Insuficiência Cardíaca**
6 de julho de 2001 – Belo Horizonte-MG	9 a 11 de junho de 2011 – Belo Horizonte-MG
I ** Congresso Brasileiro de Insuficiência Cardíaca**	**XI Congresso Brasileiro de Insuficiência Cardíaca**
28 a 30 de novembro de 2002 – Rio de Janeiro-RJ	31 de maio a 02 de junho de 2012 – Gramado-RS
**II Congresso Brasileiro de Insuficiência Cardíaca**	**XII Congresso Brasileiro de Insuficiência Cardíaca **
21 de novembro de 2003 – São Paulo-SP	6 a 8 de junho de 2013 – Porto de Galinhas-PE
**III Congresso Brasileiro de Insuficiência Cardíaca**	**XIII Congresso Brasileiro de Insuficiência Cardíaca **
25 a 27 de novembro de 2004 – Salvador-BA	7 a 9 de agosto de 2014 – Ribeirão Preto-SP
**IV Congresso Brasileiro de Insuficiência Cardíaca**	**XIV Congresso Brasileiro de Insuficiência Cardíaca**
23 a 25 de junho de 2005 – Gramado-RS	18 a 20 de junho de 2015 – Rio de Janeiro-RJ
**V Congresso Brasileiro de Insuficiência Cardíaca **	**XV Congresso Brasileiro de Insuficiência Cardíaca **
06 a 08 de julho de 2006 – Goiânia-GO	11 a 13 de agosto de 2016 – Campos do Jordão-SP
**VI Congresso Brasileiro de Insuficiência Cardíaca**	**XVI Congresso Brasileiro de Insuficiência Cardíaca**
28 a 30 de junho de 2007 – Fortaleza-CE	11 a 13 de maio de 2017 – Gramado-RS
**VII Congresso Brasileiro de Insuficiência Cardíaca **	X **VII Congresso Brasileiro de Insuficiência Cardíac** a
26 a 28 de junho de 2008 – Búzios-RJ	28 a 30 de junho de 2018 – Goiânia-GO
V **III Congresso Brasileiro de Insuficiência Cardíaca**	**XVIII Congresso Brasileiro de Insuficiência Cardíaca **
11 a 13 de junho de 2009 – São Paulo-SP	8 a 10 de agosto de 2019 – Fortaleza-CE
I **X Congresso Brasileiro de Insuficiência Cardíaca **	**Heart Failure Summit Brazil 2020 (DIGITAL)**
10 a 12 de junho de 2010 – Curitiba-PR	19 de setembro de 2020

Cumprindo seu papel científico, o DEIC tem seu importante projeto de Diretrizes e Atualizações, com o objetivo de demonstrar estratégias e propor recomendações baseadas em evidências. A primeira Diretriz de IC, na forma de um consenso, foi publicada em 1992, em uma fase antes da formação do DEIC, realizada em São Paulo sob a coordenação do estimado mestre Dr. Michel Batlouni ( Quadro 3 ).

**Quadro 3:** – Consenso e Diretrizes

- Consenso brasileiro para o tratamento da insuficiência cardíaca – 1992
- Diretrizes e atualizações de insuficiência cardíaca: 1999, 2002, 2005, 2009, 2012, 2018
- Diretrizes de transplante cardíaco: 1999, 2010, 2018
- Diretriz de cardio-oncologia: 2011
- Diretriz de miocardites e pericardite: 2013
- Diretriz de insuficiência cardíaca e transplante cardíaco, no feto, na criança e em adultos com cardiopatia congênita: 2014
- Diretriz de assistência circulatória mecânica: 2016

Destaca-se, aqui, a publicação em 2014, do I Registro Brasileiro de Insuficiência Cardíaca – Aspectos Clínicos, Qualidade Assistencial e Desfechos Hospitalares – BREATHE, organizado pelo Professor Denilson Campos de Albuquerque, projeto que traçou um panorama da IC em pacientes hospitalizados nas diversas regiões do país, identificando a aderência da incorporação de métodos diagnósticos e intervenções terapêuticas. Atualmente, está em curso o Registro Brasileiro da Síndrome de Takotsubo, liderado pelo professor Marcelo Westerlund Montera.

O DEIC tem, de forma contemporânea, ampliado sua abrangência técnico-científica, atuando na IC crônica, na IC aguda (sala de emergência/unidade cardiointensiva), na IC avançada (transplante cardíaco/suporte circulatório mecânico), na IC na criança e adolescente e também nas miocardiopatias. Na área das miocardiopatias tivemos o pioneirismo dos professores Marco Aurélio Dias, Francisco Manes Albanesi, Raul Carlos Pareto Junior, Antonio Carlos Pereira Barreto e Charles Mady, que contribuíram para a formação de líderes na IC.

Nos últimos 5 anos, foram criados em importantes áreas temáticas os grupos de estudo: Grupo de Estudos de Transplante Cardíaco e Assistência Circulatória Mecânica (GETAC), Grupo de Estudos de IC na Criança e em Adultos Portadores de Cardiopatia Congênita (GEICPED) e Grupo de Estudos em Miocardiopatias (GEMIC). Dessa forma, estamos nos organizando dentro da moderna visão de construir um ecossistema para colaboração e cooperação inter e multidisciplinar em diferentes subáreas, o que tem sido uma tendência nas sociedades internacionais de IC: HFA-ESC – https://www.escardio.org/Sub-specialty-communities/Heart-Failure-Association-of-the-ESC-(HFA) – e HFSA (https://hfsa.org/). ( Quadro 4 )

**Quadro 4:** – Programas de Residência Médica e Cursos de Especialização em IC Avançada e Transplante Cardíaco

1. Instituto Dante Pazzanese de Cardiologia
Curso de Aprimoramento em Transplante de Coração em Adultos
2. Sociedade Beneficente Israelita Brasileira Hospital Albert Einstein
Curso de Aprimoramento em Transplante e Insuficiência Cardíaca
3. Instituto do Coração (Incor) – HC-FMUSP
-Programa de complementação especializada – PCE: Insuficiência Cardíaca Congestiva e Dispositivos de Assistência Ventricular
-Residência Médica em Transplante de Coração
4. Universidade Federal de São Paulo – UNIFESP
Residência Médica da Escola Paulista de Medicina – Ano opcional: Transplante Cardíaco
5. Hospital de Clínicas de Porto Alegre
Residência Médica – Ano adicional: Transplante de Coração
6. Instituto de Cardiologia do Rio Grande do Sul/Fundação Universitária de Cardiologia
Residência Médica – Ano adicional para Capacitação em Transplante Cardíaco
7. Instituto de Medicina Integral Professor Fernando Figueira – IMIP
Programa de Complementação Especializada – COMESP na área de Transplante de
Coração e Insuficiência Cardíaca Avançada

Em 2004, foi idealizado o GEIC Jovem, buscando incentivar o desenvolvimento científico e associativo dos jovens cardiologistas interessados na IC. A primeira reunião realizada no Congresso Brasileiro de Insuficiência Cardíaca em Salvador (2004), desde então, vem se transformando a cada ano e agregando novas lideranças, e agora trazendo como foco a inovação e o empreendedorismo aos nossos congressistas.

Reafirmando seu papel social, com ações comunitárias e em políticas de saúde, durante o 73º Congresso Brasileiro de Cardiologia (2018), o presidente do DEIC, Salvador Rassi, e o diretor científico, Evandro Tinoco Mesquita, oficializaram o Dia Nacional de Alerta contra a Insuficiência Cardíaca,^3^ comemorado no dia 9 de julho. A data foi escolhida por ser o dia do nascimento de Carlos Chagas, o nosso patrono. Esse primeiro “cardiologista moderno” confirma a citação do saudoso professor Nelson Botelho, pois usou a visão translacional, aproximando a bancada do leito e conectando um olhar sobre a saúde populacional na doença de Chagas. Além disso, criamos a comenda Carlos Chagas do DEIC destinada aos colegas que se destacam nas áreas de ensino/educação, assistência, inovação, atividades e associativismo. Em parceria com a Rede Brasileira de Insuficiência Cardíaca (REBRIC), trabalhamos não somente com o alerta contra a IC, mas também com a aplicação da literacia, construindo ferramentas para o autocuidado e melhora dos desfechos clínicos (https://www.rebric.com.br/).

A próxima década nos traz novos desafios: primeiro, consolidarmos os caminhos para uma nova área de atuação na cardiologia – o especialista em IC. Na presente década, várias iniciativas, alinhadas à visão contemporânea já bem estabelecida em outros países, foram criadas com o objetivo de promover e capacitar especialistas em IC, garantindo a formação com qualidade técnica e científica.

Além disso, um olhar mais amplo sobre prevenção na IC envolve a compreensão do modelo de doença cardiometabólica crônica,^4^ passando a ser fundamental unir hipertensão arterial sistêmica, obesidade, dislipidemia e diabetes melito na gênese, progressão e tratamento da IC. E, por fim, a necessidade do cuidado integral da IC, cooperando medicina da família, geriatras, internistas, intensivistas e paliativistas. Ao lado disso, os avanços da inteligência artificial,^5^ da medicina digital e da genômica vão construindo uma medicina cardiovascular personalizada na IC que transformarão os conceitos de prevenção, diagnóstico e tratamento, conforme tem sido desenvolvido na amiloidose cardíaca e nas miocardiopatias hereditárias. A pandemia por COVID-19 reforça o conceito de cardiovigilância,^6 , 7^ pois estudos que utilizaram a ressonância cardíaca identificaram que, mesmo em pessoas sem sintomas, há um grau de agressão ao coração e que deverá ser estudado quanto ao risco futuro do desenvolvimento de miocardiopatia dilatada e insuficiência cardíaca sintomática.

**Figura 1:**
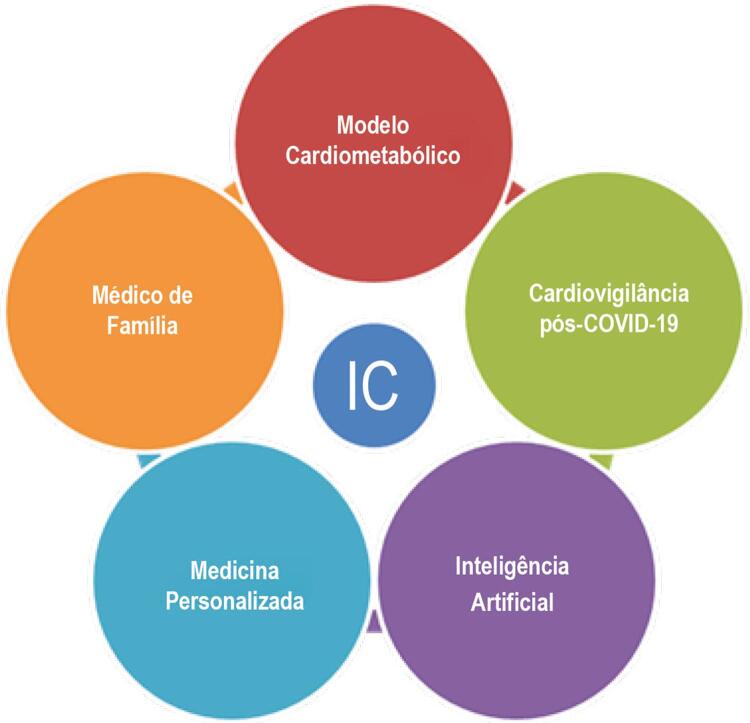
Desafios do DEIC 2020-30

Para comemorar os 20 anos de história do DEIC, reunimos as conquistas de nossa brilhante trajetória e destacamos os cardiologistas, cujo trabalho e dedicação foram responsáveis pela excelência e pelo sucesso do departamento. É uma grande honra resgatar e reverenciar o passado, vislumbrando um futuro, com o desafio de buscar novas ideias e de se renovar. ( Figura 1 ).
