# Supplementary material for: Os Desafios da Insuficiência Cardíaca Ontem, Hoje e Amanhã, e os 20 Anos do DEIC
Source: Arq Bras Cardiol. 2021 Feb 19;116(2):359–62. [Article in Portuguese] doi: 10.36660/abc.20201200 (PMC7909977; doi:10.36660/abc.20201200)
Supplement: Translation

# Translation: The Challenges of Heart Failure Yesterday, Today and Tomorrow and the 20 Years of DEIC

**Authors:** Evandro Tinoco Mesquita, Ana Paula Mendes, Lidia Moura, José Albuquerque de Figueiredo, Fabiana G. Marcondes-Braga, Fernando Bacal, Maria da Consolação Vieira Moreira, Nadine Oliveira Clausell

**Affiliations:** 1 Brasil Universidade Federal Fluminense Hospital Universitário Antônio Pedro, Niterói, RJ - Brasil; 2 Brasil Pontifícia Universidade Católica do Paraná, Curitiba, PR - Brasil; 3 Brasil Universidade Federal do Maranhão, São Luís, MA - Brasil; 4 Brasil Instituto do Coração (InCor), Hospital das Clínicas, Faculdade de Medicina, Universidade de São Paulo, São Paulo, SP - Brasil; 5 Brasil Universidade Federal de Minas Gerais Faculdade de Medicina, Belo Horizonte, MG - Brasil; 6 Brasil Hospital de Clínicas de Porto Alegre - Serviço de Cardiologia, Porto Alegre, RS – Brasil

**Keywords:** Heart Failure/trends, Demographic Aging, Translational Medical Research, Guidelines, Scientific Production

The Department of Heart Failure of the Brazilian Society of Cardiology (DEIC) completes 20 years in 2020 and represents a robust legacy of scientific activities and associations of Brazilian cardiology.

Its foundation represented an important milestone in the fight against heart failure (HF), a complex progressive and often fatal clinical syndrome. HF should be addressed in a multidisciplinary way, supported by translational science and good practices (clinical guidelines and protocols), involving patients, families, caregivers, managers and the whole society, in view of its social impacts in Brazil and in the world.

With population aging and increased survival of patients with cardiovascular diseases, the prevalence of HF is increasing globally, with about 26 million people affected worldwide, in addition to thousands of undiagnosed cases.^1^HF is the leading cause of hospitalization in the world and this results in an overload at all levels of care. It is estimated that HF affects approximately 2.5 million people in Brazil and a recent study has revealed its financial impact in Brazil, with an estimated expenditure of BRL 22.1 million/US$ 6.8 million in 2015.^2^Besides, the study revealed a substantial loss of well-being. Of the 521,941 years of life lost adjusted for disability, adjusted for comorbidities, there are 270,806 years of healthy life lost due to disability and 251,941 years of life lost as a result premature death.

DEIC was created under the leadership of Professor Maria da Consolação Vieira Moreira and had the support of Professor Gilson Soares Feitosa, then president of the Brazilian Society of Cardiology (1999–2001), mobilizing leaders from all over Brazil, particularly the professor Edimar Alcides Bocchi, leading the Heart Failure Study Group (GEIC) in 2000. On July 6, 2001, chaired by Professor Maria da Consolação, the 1^st^Brazilian Symposium on Heart Failure was held in Belo Horizonte during the 12_th_Congress of Society of Cardiology of Minas Gerais ( Chart 1 ).

**Chart 1:** – GEIC/DEIC Presidents.

First GEIC Board	GEIC/DEIC Presidents
**President:** Edimar Alcides Bocchi	**2000-2001** : Edimar Alcides Bocchi
**Vice President:** Denílson Campos Albuquerque	**2002-2003:** Edimar Alcides Bocchi
**Secretary:** Fábio Vilas-Boas Pinto	**2004-2005:** Fábio Vilas-Boas Pinto
**Scientific Director:** Maria da Consolação V Moreira	**2006-2007:** Nadine Oliveira Clausell
**Members of the Scientific Committee:**	**2008-2009:** Marcelo Westerlund Montera
Evandro Tinoco Mesquita	**2010-2011:** Fernando Bacal
Dirceu Rodrigues de Almeida	**2012-2013:** João David de Souza Neto
Fernando Bacal	**2014-2015:** Dirceu Rodrigues de Almeida
Marco Aurélio Silva ( *in memoriam* )	**2016-2017:** Luis Eduardo Paim Rohde
Nadine Oliveira Clausell	**2018- 2019:** Salvador Rassi
Salvador Rassi	**2020-2021:** Evandro Tinoco Mesquita

With a successful history, built by HF leaders in Brazil, due to the growing number of members and relevance in scientific productivity, GEIC gradually became the Department of Heart Failure (DEIC), finally created in 2011, in the administration of Professor Fernando Bacal. This important fact occurred during the 10^th^Brazilian Congress of Heart Failure, celebrating 11 years since the foundation of GEIC, in the city of Belo Horizonte ( Chart 1 ).

Since its foundation, congresses of high scientific quality and international exchange have been held annually, providing the Brazilian medical community with the improvement of the state of the art of multidisciplinary care and treatment of HF. In the last congresses, we have had more than 1,000 registrants and about 200 papers have been presented, allowing exchanges of experiences with renowned specialists from various locations, from Brazil and the world. In 2020, due to the impacts of the pandemic of the new coronavirus, DEIC, in a revolutionary way, held a virtual congress — Heart Failure Summit Brazil 2020 — to present and discuss the main advances that, in the last 12 months, transformed the HF and will be reasons for changes to our HF Guideline due to be released in the first half of 2021 ( Chart 2 ).

**Chart 2:** – GEIC/DEIC Symposia and Congresses.

**1st Brazilian Symposium on Heart Failure**	**10th Brazilian Congress of Heart Failure**
July 6, 2001 - Belo Horizonte MG	June 9 to 11, 2011 — Belo Horizonte MG
**1st Brazilian Symposium on Heart Failure**	**11th Brazilian Congress of Heart Failure**
November 28 to 30, 2002 - Rio de Janeiro RJ	May 31 to June 2, 2012 — Gramado RS
**2nd Brazilian Symposium on Heart Failure**	**12th Brazilian Congress of Heart Failure**
November 21, 2003 - São Paulo SP	June 6 to 8, 2013 — Porto de Galinhas PE
**3rd Brazilian Symposium on Heart Failure**	**13th Brazilian Congress of Heart Failure**
November 25 to 27, 2004 - Salvador BA	August 7 to 9, 2014 — Ribeirão Preto SP
**4th Brazilian Symposium on Heart Failure**	**14th Brazilian Congress of Heart Failure**
June 23 to 25, 2005 — Gramado RS	**J** une 18 to 20, 2015 — Rio de Janeiro RJ
**5th Brazilian Congress of Heart Failure**	**15th Brazilian Congress of Heart Failure**
July 06 to 08, 2006 — Goiânia GO	August 11 to 13, 2016 — Campos do Jordão SP
**6th Brazilian Congress of Heart Failure**	**16th Brazilian Congress of Heart Failure**
June 28 to 30, 2007 — Fortaleza CE	May 11 to 13, 2017 — Gramado RS
**7th Brazilian Congress of Heart Failure**	**17th Brazilian Congress of Heart Failure**
June 26 to 28, 2008 — Búzios RJ	June 28 to 30, 2018 — Goiânia GO
**8th Brazilian Congress of Heart Failure**	**18th Brazilian Congress of Heart Failure**
June 11 to 13, 2009 - São Paulo SP	August 8 to 10, 2019 — Fortaleza CE
**9th Brazilian Congress of Heart Failure**	**Heart Failure Summit Brazil 2020 (DIGITAL)**
June 10 to 12, 2010 — Curitiba PR	September 19, 2020

Fulfilling its scientific role, DEIC has an important project of Guidelines and Updates, intended to demonstrate strategies and propose evidence-based recommendations. The first HF Guideline, in the form of a consensus, was published in 1992, before the DEIC was created. It was published in São Paulo and was coordinated by the esteemed master Dr. Michel Batlouni ( Chart 3 ).

**Chart 3:** – Consensuses and Guidelines

- Brazilian Consensus for the Treatment of Heart Failure — 1992
- Heart Failure Guidelines and Updates: 1999, 2002, 2005, 2009, 2012, 2018
- Heart Transplant Guidelines: 1999, 2010, 2018
- Onco-Oncology Guideline: 2011
- Guideline on Myocarditis and Pericarditis: 2013
- Guideline on Heart Failure and Heart Transplantation in Fetuses, Children and Adults with Congenital Heart Diseases: 2014
- Guideline on Mechanical Circulatory Assistance: 2016

In 2014, the 1^st^Brazilian Registry of Heart Failure — Clinical Aspects, Quality of Care and Hospital Outcomes — BREATHE was published. It was organized by Professor Denilson Campos de Albuquerque and outlined a picture of HF in hospitalized patients around the country, identifying the incorporation of diagnostic methods and therapeutic interventions. The Brazilian Registry of Takotsubo Syndrome, led by Professor Marcelo Westerlund Montera, is currently underway.

DEIC has taken a contemporary stance and expanded its technical and scientific scope, working on chronic HF, acute HF (emergency room/cardiovascular intensive care unit), advanced HF (heart transplantation/mechanical circulatory support), HF in children and adolescents and cardiomyopathies. In Cardiomyopathy, we had the pioneering spirit of teachers Marco Aurélio Dias, Francisco Manes Albanesi, Raul Carlos Pareto Junior, Antonio Carlos Pereira Barreto, Charles Mady, who helped train leaders in HF.

Over the last five years, the following study groups were created in important thematic areas: GETAC (Heart Transplant and Mechanical Circulatory Assistance Study Group), GEICPED (Study Group on HF in Children and Adults with Congenital Heart Diseases) and GEMIC (Cardiomyopathy Study Group). From a modern perspective, we are organizing ourselves to build an ecosystem for inter and multidisciplinary collaboration and cooperation in different sub-areas, which has been a trend in the following international HF societies: HFA-ESC (https://www.escardio.org/Sub-specialty-communities/Heart-Failure-Association-of-the-ESC-(HFA) and HFSA (https://hfsa.org/) ( Chart 4 ).

**Chart 4:** – Medical Residency Programs and Specialization Courses in Advanced HF and Heart Transplantation

1. Instituto Dante Pazzanese de Cardiologia
Continuing Education on Heart Transplant in Adults
2. Sociedade Beneficente Israelita Brasileira Hospital Albert Einstein
Continuing Education on Transplant and Heart Failure
3. Instituto do Coração (Incor) - HCFMUSP
- Specialized complementation program: Congestive Heart Failure and Ventricular Assist Devices
- Heart Transplant Residency
4. Universidade Federal de São Paulo - UNIFESP
Medical Residency at Escola Paulista de Medicina - Optional Year: Heart Transplant
5. Hospital de Clínicas de Porto Alegre
Medical Residency - Additional Year: Heart Transplant
6. Instituto de Cardiologia do Rio Grande do Sul/ Fundação Universitária de Cardiologia
Medical Residency - Additional year for heart transplant education
7. Instituto de Medicina Integral Professor Fernando Figueira – IMIP
Specialized Complementation Program — COMESP. Heart Transplant and Advanced Heart Failure.

In 2004, the GEIC for the Youth was created to encourage the scientific and associative development of young cardiologists interested in HF. The first meeting held at the Brazilian Congress of Heart Failure in Salvador (2004), since then, has been changing each year and adding new leaders and now bringing innovation and entrepreneurship to our attendees.

Reaffirming its social role, with community actions and health policies, in the 73^rd^Brazilian Congress of Cardiology (2018), DEIC president Salvador Rassi and scientific director Evandro Tinoco Mesquita made official the National Day of Alert against Heart Failure,^3^celebrated on the 9^th^of July. The date was chosen because it was the day of birth of Carlos Chagas, our patron. This first “modern cardiologist” confirms the quote from the late Professor Nelson Botelho, as he used a translational view bringing the bench closer to the bed and connecting a look at population health in Chagas disease. Besides, we created the Carlos Chagas commendation from DEIC for colleagues outstanding in teaching/education, assistance, innovation, science and associations. In collaboration with REBRIC (Brazilian Network of Heart Failure) we work not only with alerts against HF, but also with the application of literacy, building tools for self-care and improvement of clinical outcomes (https://www.rebric.com.br/).

The next decade brings us new challenges: firstly, cementing the paths to a new area of expertise in cardiology — the HF specialist. In the present decade, in line with a contemporary view well established in other countries, several initiatives were introduced to promote and train HF specialists, ensuring education with technical and scientific quality.

In addition, a broader look at prevention in HF involves understanding the model of chronic cardiometabolic disease,^4^making it essential to combine systemic arterial hypertension, obesity, dyslipidemia and diabetes mellitus in the genesis, progression and treatment of HF. Lastly, the need for comprehensive care of HF, involving general practitioners, geriatricians, internists, intensivists and palliative care specialists. Besides, advances in artificial intelligence,^5^digital medicine and genomics are building personalized cardiovascular medicine in HF that will transform the concepts of prevention, diagnosis and treatment, as it has been developed in cardiac amyloidosis and hereditary cardiomyopathies. The COVID-19 pandemic further establishes the concept of cardiovascular surveillance,^6 , 7^as studies that used cardiac resonance imaging found that, even in people without symptoms, there is a degree of aggression to the heart that should be studied regarding the future risk of developing dilated cardiomyopathy and symptomatic heart failure.

To celebrate the 20 years of DEIC, we gathered the achievements of our brilliant story and applauded the cardiologists, whose work and dedication were responsible for the excellence and success of the department. It is a great honor to revive and revere the past, envisioning a future with the challenge of producing new ideas and renewing ourselves ( Figure 1 ).

**Figure 1:** – Challenges of DEIC 2020–30
